# Timing of Initiation of Palliative Chemotherapy in Asymptomatic Patients with Metastatic Pancreatic Cancer: An International Expert Survey and Case-Vignette Study

**DOI:** 10.3390/cancers15235603

**Published:** 2023-11-27

**Authors:** Simone Augustinus, Hanneke W. M. van Laarhoven, Geert A. Cirkel, Jan Willem B. de Groot, Bas Groot Koerkamp, Teresa Macarulla, Davide Melisi, Eileen M. O’Reilly, Hjalmar C. van Santvoort, Tara M. Mackay, Marc G. Besselink, Johanna W. Wilmink

**Affiliations:** 1 Department of Surgery, Location University of Amsterdam, Amsterdam University Medical Center, 1105 Amsterdam, The Netherlands; s.augustinus@amsterdamumc.nl (S.A.); m.g.besselink@amsterdamumc.nl (M.G.B.); 2 Cancer Center Amsterdam, 1105 Amsterdam, The Netherlands; 3 Department of Medical Oncology, Location University of Amsterdam, Amsterdam University Medical Center, 1105 Amsterdam, The Netherlands; 4 Department of Medical Oncology, University Medical Center Utrecht, Utrecht University, 3584 Utrecht, The Netherlands; 5 Department of Medical Oncology, Isala Oncology Center, 8025 Zwolle, The Netherlands; 6 Department of Surgery, Erasmus MC Cancer Institute, 3015 Rotterdam, The Netherlands; 7 Department of Medical Oncology, Vall d’Hebron Unveristy Hospital, Vall d’Hebron Institute of Oncology (VHIO), 08035 Barcelona, Spain; 8 Digestive Molecular Clinical Oncology Unit, Univeristy of Verona, 37134 Verona, Italy; 9 Department of Medical Oncology, Memorial Sloan Kettering Cancer Center, New York, NY 10065, USA; 10 Department of Surgery, Regional Academic Cancer Center Utrecht, UMC Utrecht Cancer Center, St. Antonius Hospital Nieuwegein, Utrecht University, 3584 Utrech, The Netherlands

**Keywords:** expert survey, case-vignette, pancreatic cancer, asymptomatic, timing of chemotherapy

## Abstract

**Simple Summary:**

The use of imaging, in general and during follow-up after resection of pancreatic cancer, is increasing. Consequently, the number of asymptomatic patients diagnosed with metastatic pancreatic cancer (mPDAC), both at initial diagnosis and during the diagnosis of recurrent disease, is increasing. In these patients, palliative systemic therapy is the only (tumor-directed) therapy and, hence, is often immediately initiated. However, delaying therapy until symptoms occur may preserve quality of life and avoid therapy-related toxicity, but the impact on survival of this approach is unknown. Using an online survey sent to all first and last authors of published trials on mPDAC and to medical oncologists of the Dutch Pancreatic Cancer group, this study aimed to gain insight into the current perspectives and clinical decision-making of experts. Overall, 78 of 291 (27%) medical oncologists from 15 countries responded. Two-thirds of respondents (63%) preferred an immediate initiation of chemotherapy following diagnosis. In 3/9 case-vignettes, delayed treatment was favored in specific clinical contexts (i.e., patient with only one small lung metastasis, significant comorbidities, and higher age). Respondents from the Netherlands, as well as medical oncologists with fewer years of experience, more often favored delayed treatment. Although the response rate was limited, in this increasing group of asymptomatic patients with mPDAC, immediate treatment is most often preferred, although in specific clinical contexts (i.e., limited metastatic disease, more comorbidities, and higher age), delaying treatment until symptoms occur is considered.

**Abstract:**

**Background**: The use of imaging, in general, and during follow-up after resection of pancreatic cancer, is increasing. Consequently, the number of asymptomatic patients diagnosed with metastatic pancreatic cancer (mPDAC) is increasing. In these patients, palliative systemic therapy is the only tumor-directed treatment option; hence, it is often immediately initiated. However, delaying therapy in asymptomatic palliative patients may preserve quality of life and avoid therapy-related toxicity, but the impact on survival is unknown. This study aimed to gain insight into the current perspectives and clinical decision=making of experts regarding the timing of treatment initiation of patients with asymptomatic mPDAC. **Methods**: An online survey (13 questions, 9 case-vignettes) was sent to all first and last authors of published clinical trials on mPDAC over the past 10 years and medical oncologists of the Dutch Pancreatic Cancer Group. Inter-rater variability was determined using the Kappa Light test. Differences in the preferred timing of treatment initiation among countries, continents, and years of experience were analyzed using Fisher’s exact test. **Results**: Overall, 78 of 291 (27%) medical oncologists from 15 countries responded (62% from Europe, 23% from North America, and 15% from Asia–Pacific). The majority of respondents (63%) preferred the immediate initiation of chemotherapy following diagnosis. In 3/9 case-vignettes, delayed treatment was favored in specific clinical contexts (i.e., patient with only one small lung metastasis, significant comorbidities, and higher age). A significant degree of inter-rater variability was present within 7/9 case-vignettes. The recommended timing of treatment initiation differed between continents for 2/9 case-vignettes (22%), in 7/9 (77.9%) comparing the Netherlands with other countries, and based on years of experience for 5/9 (56%). **Conclusions**: Although the response rate was limited, in asymptomatic patients with mPDAC, immediate treatment is most often preferred. Delaying treatment until symptoms occur is considered in patients with limited metastatic disease, more comorbidities, and higher age.

## 1. Introduction

Pancreatic ductal adenocarcinoma (PDAC) is one of the deadliest forms of cancer [1]. Almost 50% of patients are diagnosed with metastatic disease (mPDAC) at diagnosis; this number is even higher when including patients with recurrent disease after resection [2]. For patients with mPDAC, the treatment options consist of palliative chemotherapy and/or best supportive care [2]. Palliative chemotherapy with FOLFIRINOX (5-fluorouracil, folinic acid, oxaliplatin, and irinotecan) improves overall survival from 6.8 to 11.1 months, whereas gemcitabine plus nab-paclitaxel improves overall survival from 6.7 to 8.5 months, as compared to gemcitabine monotherapy [3,4]. Furthermore, in patients with symptomatic mPDAC, palliative chemotherapy can decrease tumor burden, and thereby diminish disease symptoms, and improve quality of life [5,6]. In the case of mPDAC, deterioration of quality of life at six months after the start of chemotherapy is reported twice as often after gemcitabine than after the more impactful yet toxic FOLFIRINOX regimen (66% versus 31%) [3].

Due to an increase in the use of imaging, in general and during follow-up after resection of pancreatic cancer, the number of asymptomatic patients with advanced cancers appears to be increasing [7,8]. Structured follow-up after surgery for PDAC remains controversial; it is associated with the detection of asymptomatic recurrence [9,10,11]. Currently, NCCN (National Comprehensive Cancer Network) guidelines advise an abdominal CT scan every three to six months in the first two years after surgical resection [12]. ESMO (European Society for Medical Oncology) guidelines advise a follow-up schedule discussed with patients, but in cases of elevated preoperative serum CA 19.9, an abdominal CT scan is advised every six months [13].

In patients with asymptomatic mPDAC, the potential benefits of palliative chemotherapy must be weighed against the toxicity associated with treatment. One perspective is that delaying therapy until symptoms occur may preserve quality of life. On the other hand, a delay in therapy could negatively impact survival or result in missing the window of opportunity to initiate chemotherapy. Two reviews have been performed on the timing of treatment initiation in asymptomatic metastatic cancer, one including three randomized trials in asymptomatic patients with colorectal cancer and the other including two additional studies including patients with advanced asymptomatic ovarian and gastric cancer [14,15]. Although it is concluded that limited evidence is available, meta-analyses demonstrate no difference in overall survival among the two groups, and a potential benefit in quality of life. For pancreatic cancer, no randomized trials have been performed, but two retrospective studies found no association between time to treatment initiation and survival in advanced pancreatic cancer [16,17]. 

Due to the limited evidence on the timing of initiation of chemotherapy in asymptomatic patients with mPDAC, clinical decision-making is mostly based on local expertise and preference. Meanwhile, there are few insights into what drives decision-making in clinical practice. This study aims to gain insight into the current opinions and clinical decision-making of medical oncologists regarding the timing of initiation of systemic therapy in asymptomatic patients with mPDAC. 

## 2. Methods

### 2.1. Study Design

Between April 2021 and November 2021, all first and senior authors of published clinical trials on mPDAC over the past 10 years (PubMed) and medical oncologists of the Dutch Pancreatic Cancer Group (DPCG) received an email invitation and three reminders to participate in an online survey regarding the timing of onset of systemic treatment in patients with asymptomatic mPDAC. The survey questions were developed, revised, and improved by an international multidisciplinary writing committee (i.e., the authors). The writing committee included leading experts in the field of pancreatic cancer treatment to ensure the scientific quality of the survey. The survey was conducted anonymously using Google Forms, but responders were given the opportunity to leave their contact information. The medical ethical committee of the Amsterdam UMC assessed the study and confirmed that the Medical Research Involving Human Subjects Act (WMO) did not apply to the study. Subsequently, the need for informed consent was waived, as no patients were involved. This study followed the AAPOR guidelines for survey studies [18].

### 2.2. Survey and Case-Vignettes

The survey contained 13 questions followed by nine clinical case-vignettes pertaining to the timing of treatment in patients with asymptomatic mPDAC (see File S1). The basic clinical scenario for all case-vignettes was as follows: *a 60-year-old asymptomatic patient with no significant comorbidities (WHO 0), one year after pancreatoduodenectomy for PDAC, was diagnosed with one liver metastasis during routine follow-up which was pathologically proven.* Only one aspect was changed per case-vignette (indicated in bold print). In the first four case-vignettes, the location and number of metastases differed; in the following two case-vignettes, the age and comorbidities differed. Case-vignette 7 included a patient with newly diagnosed mPDAC in contrast to recurrence after resection. In the last two case-vignettes, treatment initiation had been already delayed for six weeks, and imaging demonstrated the progression of the disease. In these scenarios, the location and number of metastases also differed. 

Respondents were asked if they would advise immediate treatment—start directly with chemotherapy (option A); delay treatment initiation—hold off on chemotherapy until emergence of symptoms (option B); delay treatment—hold off chemotherapy until disease progression on imaging (option C); delayed treatment—hold off on chemotherapy until symptoms and/or disease progression on imaging (option D); and whichever comes first, or other (option E).

### 2.3. Data Collection

The preferred treatment options in the case-vignettes were subsequently categorized as either immediate treatment, delayed treatment, or other. When respondents filled in the “other” field in the survey, but a preference for immediate or delayed treatment was implicated, that response was recoded to the corresponding group. Recoding was performed by the first author (SA) and checked by another author (TM). 

### 2.4. Statistical Analysis

Results are reported as proportions for binary or categorical variables, and as means with standard deviation (SD) or medians with interquartile range (IQR) for continuous variables, when appropriate. Inter-rater variability was calculated with the Light’s Kappa (Kappa for more than two raters) and defined as the K-value. Interpretation of the K-value was based on Kappa value interpretation by Landis and Koch as follows: poor (≤0), slight (0.01–0.20), fair (0.21–0.40), moderate (0.41–0.60), substantial (0.61–0.80) or perfect (0.81–1.00) [19]. A Fisher’s exact test was used to compare the differences in preferred treatment among the different groups of respondents. For statistical analysis, RStudio (version 4.0.3) was used. A two-tailed *p*-value of <0.05 was considered statistically significant. 

## 3. Results

### 3.1. Characteristics of Respondents

Overall, 78 of the 291 (27%) invited medical oncologists from 15 countries responded. All respondents completed the survey in its totality. The characteristics are depicted in Table 1. The majority of respondents were from Europe (62%), worked in an academic hospital (73%), and the mean age was 52 years (SD 10). Overall, 62% of respondents reported that structured follow-up following resection for PDAC was routinely performed at the hospitals where they worked (for example, imaging every three months after resection). For 24% of respondents, structured follow-up was only conducted in the context of clinical trials.

Respondents treated a varying number of patients with chemotherapy for PDAC (all stages) (Table 1); the majority of respondents treated 21 to 50 patients per year (n = 34, 44%). In general, the preferred type of palliative chemotherapy for metastatic PDAC, specifically, was FOLFIRINOX (67%), followed by gemcitabine plus nab-paclitaxel (18%), or a different treatment in the context of a clinical trial (15%). None of the respondents preferred gemcitabine monotherapy. Most of the respondents treated less than 10 asymptomatic patients with mPDAC per year (n = 49, 64%), of which six respondents treated no asymptomatic mPDAC at all (8%). 

### 3.2. Perspectives on Timing of Treatment of Asymptomatic Patients with mPDAC

**Figure 1:**
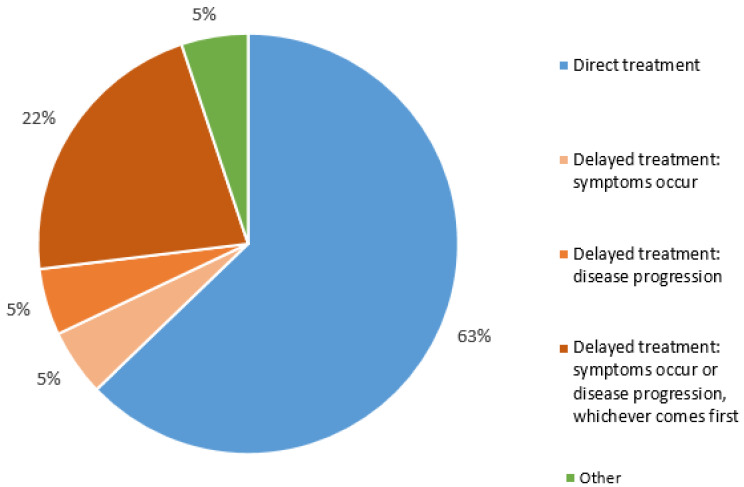
Perspectives on systemic treatment in asymptomatic patients with mPDAC.

The majority of participants (n = 49/78, 63%) answered that the best time point to initiate systemic treatment in patients with asymptomatic PDAC was immediately following the diagnosis. The respondents preferring delayed treatment would consider starting when symptoms occurred or in the setting of objective disease progression (n = 17/78, 22%), only once symptoms occurred (n = 4/78, 5%), or in the setting of objective signs of disease progression (radiologic, tumor marker) after the first diagnosis of mPDAC (n = 4/78, 5%, Figure 1). Almost all respondents (n = 77/78, 99%) indicated that financial incentives did not play a role in the decision regarding when to commence systemic treatment. One respondent indicated that financial incentives did play a role. A randomized trial to assess the optimal timing of starting systemic therapy in patients with asymptomatic mPDAC was justified by 54% of respondents.

### 3.3. Case-Vignettes

In six of nine case-vignettes (66%), immediate treatment was preferred. In almost all case-vignettes where the patient was 60 years old and had no comorbidities, immediate treatment was preferred regardless of the disease volume or location of metastases (Table 2). These case-vignettes involved a patient with recurrent disease and one liver metastasis (case-vignette 1: 49%), recurrent disease and six lung metastases (case-vignette 3: 73%), recurrent disease and three liver and three lung metastases (case-vignette 4: 89%), and primary diagnosis of PDAC with one liver metastasis (case-vignette 7: 72%). An exception was the case-vignette where the patient had recurrent disease and one small lung metastasis (detected on CT and pathologically confirmed), in which delayed treatment was preferred by the respondents (case-vignette 2: 49%). In the case-vignettes where the patient was of higher age (case-vignette 5) or suffered significant comorbidities (case-vignette 6), delayed treatment was also preferred. In these case-vignettes, the number of respondents choosing the “other” option also increased, by 24% and 30%, respectively. The respondents who filled in “other” mostly preferred best supportive care, local treatment, or shared decision-making. In both case-vignettes where disease progression was seen after six weeks (case-vignettes 8 and 9), regardless of the degree of progression, nearly all respondents (>90%) chose immediate treatment.

### 3.4. Inter-Rater Variability

The inter-rater variability varied among case-vignettes. There was “slight agreement” among raters in four case-vignettes, “fair agreement” in two case-vignettes, “substantial agreement” in one case-vignette, and “perfect agreement” in two case-vignettes (Table 2). Perfect agreement was present in the case-vignettes where patients had disease progression after six weeks of delayed treatment.

### 3.5. Variation in the Timing of Treatment Initiation

Significant variability in the preferred timing of treatment among continents was present in only two of nine case-vignettes, namely in case-vignette 1 and 2 (*p* = 0.012, *p* = 0.032). In both case-vignettes, most respondents in Europe preferred delayed treatment, compared to direct treatment in the USA and the Asia–Pacific (Table S1). Variability in the preferred timing between the Netherlands and other countries was present in seven of nine case-vignettes (Table S2). In all case-vignettes, the Netherlands had a higher percentage of respondents opting for delayed treatment and a lower percentage opting for immediate treatment. When comparing the responses from medical oncologists with different years of experience (i.e., <5 years, 5–10 years, >10 years), there was variability in the preferred timing of treatment in five of nine case-vignettes (Table S3). In all of these case-vignettes, the percentage of delayed treatment was highest for the medical oncologists with less than five years of experience. 

## 4. Discussion

This first survey investigating expert medical oncologists’ perspectives regarding the timing of treatment initiation of palliative chemotherapy in asymptomatic patients with mPDAC demonstrated that immediate treatment was mostly preferred (63%). In one-third of case-vignettes, delayed treatment initiation was favored. A significant degree of inter-rater variability was present within most (7/9) case-vignettes. Medical oncologists with less than five years of experience and those from the Netherlands more often preferred delayed treatment.

The results of this study suggest that, in patients with apparently less aggressive disease biology (i.e., one small lung metastasis), delayed chemotherapy was more often the preferred option. Regardless of symptoms, patients affected by disease recurrence at multiple sites after resection have the worst overall survival (7.2 months) compared to patients with lung-only metastases with the best overall survival (15.8 months) [20]. On the contrary, some evidence suggests that, in patients with more favorable disease biology, more intensive therapy and/or localized therapy may be justified [21]. 

In the case-vignettes that included a patient of higher age, or a patient with several comorbidities, delayed treatment was more often favored. This confirms our expectations, as previous studies have shown that these patients, in general, are less likely to receive chemotherapy [22,23]. This may be due to the fact that patients of higher age are more likely to experience treatment-related toxicity. In a study investigating patients with advanced pancreatic cancer receiving first-line chemotherapy (n = 203), elderly patients (≥70 years old) suffered a higher incidence of severe adverse events compared to younger patients (<70 years old, 50% versus 29.3%) [24]. In particular, FOLFIRINOX is considered mainly suitable for younger and relatively fit patients, as there is an increase in toxicity associated with this regimen, compared to gemcitabine +/− nab paclitaxel [25]. Nevertheless, a systematic review including five studies showed that combination chemotherapy is associated with a similar survival benefit in patients of higher age compared to younger patients [26]. Also, in the case-vignettes, when including this patient group, there was low inter-observer agreement among respondents. These findings may reflect the high percentage of respondents that favored neither immediate nor delayed treatment (24.4% and 29.5%, respectively) but considered best supportive care, local treatment or shared decision-making as the best option. Therefore, particularly in the older patient group or with the patients with significant comorbidities, shared decision-making considering different treatment strategies, such as best supportive care, is of great importance. 

Moreover, in this study, both patients with mPDAC at initial diagnosis and diagnosed during disease recurrence were included. In both treatment groups, palliative systematic therapy or best supportive care were the only treatment options. The basic case included a patient diagnosed with recurrent disease; however, case 7 included a patient with mPDAC as the primary diagnosis. Within both these cases, immediate treatment was preferred, emphasizing that these two groups can be compared. However, the differences among these two groups have to be taken into account when interpreting the results.

In only three of nine case-vignettes, there was a near complete consensus among respondents, but in all six other case-vignettes, there was a relatively low degree of inter-observer agreement. This may reflect the lack of evidence on this topic and suggests that decision-making is mainly based on local expertise and personal beliefs. Currently, the Dutch randomized trial (TIMEPAN) is enrolling asymptomatic patients with mPDAC to immediate or delayed chemotherapy (ClinicalTrials.gov Identifier: NCT04897854) [27]. The primary endpoint is quality-adjusted survival, and the results may provide further guidance for clinicians involved in the decision-making on this topic.

Variability in decision-making among practitioners in different countries was apparent in two of nine case-vignettes. When comparing the Netherlands versus the rest of the world, however, there was variability in seven of nine case-vignettes, indicating a stronger preference for delayed treatment in the Netherlands. This might be due to several reasons. First, Dutch medical oncologists may be more familiar with delayed treatment due to the TIMEPAN trial, which is currently ongoing in the Netherlands [27]. Second, in general, patients in the Netherlands have a lower chance of receiving palliative chemotherapy compared to the USA [28]. A retrospective study comparing the proportion of patients of higher age receiving palliative chemotherapy for pancreatic cancer in the Netherlands with the Moffitt Cancer Center in Florida, USA, noted a significant difference (17% versus 65%) [28]. It is difficult to compare results between a nationwide analysis and a tertiary center analysis. Therefore, proportions between Dutch academic centers and the Moffitt center were also compared, and the difference was less evident (54% versus 65%). Additionally, medical oncologists with less than five years of experience preferred delayed treatment more often in this study. This might be due to the different perspective of a younger group of medical oncologists and to more focus on shared decision-making during their traineeship [29]. 

This survey should be interpreted in light of several key limitations. First, the response rate of participants was low. This may be due to selection factors for participation, which are based on the first and last authors of publications of clinical trials on mPDAC in the past 10 years; some email addresses may be outdated and, potentially, authors may no longer treat patients with PDAC. Second, as most respondents were from academic centers and from Europe (mainly the Netherlands), the results of this survey might not give an overview of the general international opinion on this topic. Third, respondents may have felt that some cases could not be pressed into simple answers, as in five of eight case-vignettes, the number of respondents that chose neither immediate nor delayed treatment was >10%. However, we chose this construct intentionally, as it forces respondents to focus on the timing of treatment instead of the type of treatment. Another notable limitation of this survey is that there are no data from randomized studies to inform the best outcomes for many of the case scenarios utilized. Therefore, the perspectives are those of the participants and there is, in these cases, no clearly validated ‘right’ or ‘wrong’ answer. Nevertheless, this survey provides a global overview of the current opinions and decision-making of medical oncologists on the timing of treatment of chemotherapy in asymptomatic patients with mPDAC.

## 5. Conclusions

In conclusion, although the response rate was limited, this is the first survey investigating the current perspectives regarding the timing of treatment initiation in asymptomatic patients with mPDAC. Immediate treatment was mostly preferred in asymptomatic patients with mPDAC; however, a substantial degree of inter-rater variability was present. In one-third of case-vignettes, delayed treatment initiation was favored (e.g., due to patient selection and medical oncologist preference). The relation between the timing of systemic treatment initiation and outcomes in patients with mPDAC is unknown and warrants further investigation.

## Figures and Tables

**Table 1:** Characteristics of survey respondents.

	All Respondents (n = 78)
Continent	
Asia–Pacific	12 (15.4%)
Europe	48 (61.5%)
USA	18 (23.1%)
Age, mean years (SD)	51.9 (SD 9.9)
Female	28 (35.9%)
Type of hospital	
Academic hospital with pancreatic surgery	53 (67.9%)
Academic hospital without pancreatic surgery	4 (5.1%)
Non-academic hospital with pancreatic surgery	10 (12.8%)
Non-academic hospital without pancreatic surgery	11 (14.1%)
Years registered as medical oncologist	
5 years or less	8 (10.3%)
6–10 years	11 (14.1%)
11 years or more	59 (75.6%)
Preferred treatment for mPDAC	
FOLFIRINOX	52 (66.7%)
Gemcitabine + nab-paclitaxel	14 (17.9%)
Gemcitabine monotherapy	0
Clinical trials	12 (15.4%)
Number of patients per year with pancreatic cancer	
None	1 (1.3%)
Less than 10 patients	7 (8.9%)
11–20 patients	15 (19.2%)
21–50 patients	34 (43.6%)
51–100 patients	12 (14.3%)
>100 patients	9 (11.5%)
Number of patients with asymptomatic pancreatic cancer	
None	6 (7.7%)
Less than 10 patients	43 (55.1%)
11–20 patients	16 (20.5%)
>20 patients	13 (16.7%)
Structured follow-up after resection	
Yes	48 (61.5%)
Only within clinical trials	19 (24.4%)
No	11 (14.1%)

Values are numbers with valid percentages unless indicated otherwise. mPDAC = metastastic pancreatic ductal adenocarcinoma, USA = United States of America, FOLFIRINOX = 5-fluorouracil, folinic acid, oxaliplatin and irinotecan.

**Table 2:** Case-vignettes.

Case-Vignettes	Age and Clinical Condition	Disease	Preferred Treatment (n = 78)		Agreement **
Case-vignette 1	60 years old No comorbidities	1 liver metastasis (recurrence)	Immediate	**38 (48.7%)**	Slight agreement (K = 0.06)
			Delayed	26 (33.3%)	
			Other	14 (17.9%)	
Case-vignette 2	60 years old No comorbidities	1 small lung metastasis (recurrence)	Immediate	23 (29.5%)	Slight agreement (K = 0.11)
			Delayed	**43 (55.1%)**	
			Other	12 (15.4%)	
Case-vignette 3	60 years old No comorbidities	6 lung metastases (recurrence)	Immediate	**57 (73.1%)**	Fair agreement (K = 0.38)
			Delayed	18 (23.1%)	
			Other	3 (3.8%)	
Case-vignette 4	60 years old No comorbidities	3 liver + 3 lung metastases (recurrence)	Immediate	**69 (88.5%)**	Substantial agreement (K = 0.68)
			Delayed	7 (9.0%)	
			Other	2 (2.6%)	
Case-vignette 5	80 years old No comorbidities	1 liver metastasis (recurrence)	Immediate	27 (34.6%)	Slight agreement (K = 0.01)
			Delayed	**32 (41.0%)**	
			Other	19 (24.4%)	
Case-vignette 6	60 years old Significant comorbidities *	1 liver metastasis (recurrence)	Immediate	18 (23.1%)	Slight agreement (K = 0.04)
			Delayed	**37 (47.4%)**	
			Other	23 (29.5%)	
Case-vignette 7	60 years old No comorbidities	1 liver metastasis (primary tumor)	Immediate	**56 (71.8%)**	Fair agreement (K = 0.33)
			Delayed	14 (17.9%)	
			Other	8 (10.3%)	
Case-vignette 8	60 years old No comorbidities	Progression from 2 to 6 liver metastasis (after 6 weeks delayed)	Immediate	**75 (96.2%)**	Perfect agreement (K = 0.85)
			Delayed	3 (3.8%)	
			Other	0	
Case-vignette 9	60 years old No comorbidities	Progression from 2 liver to 3 liver + 3 lung metastasis (after 6 weeks delayed)	Immediate	**73 (93.6%)**	Perfect agreement (K = 0.82)
			Delayed	3 (3.8%)	
			Other	2 (2.6%)	

* Type 2 diabetes and heart failure NYHA class 2; due to the comorbidities the WHO performance status is 2. ** Interpretation based on Kappa value interpretation according to Landis and Koch (1977): <0 No agreement, 0–0.20 slight agreement, 0.21–0.40 fair agreement, 0.41–0.60 fair agreement, 0.61–0.80 substantial agreement, 0.81–1.0 perfect agreement. Underscored text indicates the change in the clinical scenario compared to case 1 (the basic case). Bold numbers indicate the preferred treatment.

## Data Availability

Data can be made available upon request.

## Supplementary Materials

The following supporting information can be downloaded at: https://www.mdpi.com/article/10.3390/cancers15235603/s1, Table S1. Case vignettes and continent; Table S2. Case vignettes: Netherlands versus other countries; Table S3. Case vignettes and years of experience as a medical oncologist; File S1. Survey.
