# Supplementary material for: Timing of Initiation of Palliative Chemotherapy in Asymptomatic Patients with Metastatic Pancreatic Cancer: An International Expert Survey and Case-Vignette Study
Source: Cancers (Basel). 2023 Nov 27;15(23):5603. doi: 10.3390/cancers15235603 (PMC10705283; doi:10.3390/cancers15235603)
Supplement: Supplementary file 1 [file cancers-15-05603-s001.zip › cancers-2647468-supplementary.pdf]

SUPPLEMENT Table S1. Case vignettes and continent

Case-vignette	Preferred treatment	Europe (n = 48)	USA (n = 18)	Asia-Pacific (n = 12)	P value
Case-vignette 1	Immediate treatment	19 (39.6%)	**12 (66.7%)**	**7 (58.3%)**	0.006
	Delayed treatment	**23 (47.9%)**	1 (5.6%)	2 (16.7%)	
	Other	6 (12.5%)	5 (27.8%)	3 (25.0%)	
Case-vignette 2	Immediate treatment	10 (20.8%)	**8 (44.4%)**	**5 (41.7%)**	0.032
	Delayed treatment	**33 (68.8%)**	6 (33.3%)	4 (33.3%)	
	Other	5 (10.4%)	4 (22.2%)	3 (25.0%)	
Case-vignette 3	Immediate treatment	**32 (66.7%)**	**14 (77.8%)**	**11 (91.7%)**	0.453
	Delayed treatment	14 (29.2%)	3 (16.7%)	1 (8.3%)	
	Other	2 (4.2%)	1 (5.6%)	0	
Case-vignette 4	Immediate treatment	**41 (85.4%)**	**17 (94.4%)**	**11 (91.7%)**	0.438
	Delayed treatment	6 (12.5%)	0	1 (8.3%)	
	Other	1 (2.1%)	1 (5.6%)	0	
Case-vignette 5	Immediate treatment	12 (25.0%)	**11 (61.1%)**	**4 (33.3%)**	0.069
	Delayed treatment	**24 (50.0%)**	3 (16.7%)	**5 (41.7%)**	
	Other	12 (25.0%)	4 (22.2%)	3 (25.0%)	
Case-vignette 6	Immediate treatment	10 (20.8%)	**7 (38.9%)**	1 (8.3%)	0.192
	Delayed treatment	**26 (54.2%)**	5 (27.8%)	**6 (50.0%)**	
	Other	12 (25.0%)	6 (33.3%)	5 (41.7%)	
Case-vignette 7	Immediate treatment	**31 (64.6%)**	**15 (83.3%)**	**10 (83.3%)**	0.056
	Delayed treatment	13 (27.1%)	0	1 (8.3%)	
	Other	4 (8.3%)	3 (16.7%)	1 (8.3%)	
Case-vignette 8	Immediate treatment	**45 (93.8%)**	**18 (100%)**	**12 (100%)**	0.733
	Delayed treatment	3 (6.3%)	0	0	
	Other	0	0	0	
Case-vignette 9	Immediate treatment	**43 (89.6%)**	**18 (100%)**	**12 (100%)**	0.901
	Delayed treatment	3 (6.3%)	0	0	
	Other	2 (4.2%)	0	0	

Bold numbers indicate the preferred treatment per continent.

SUPPLEMENT Table S2. Case vignettes: Netherlands versus other countries

Case-vignette	Preferred treatment	Netherlands (n = 30)	Others (n = 48)	P value
Case-vignette 1	Immediate treatment	4 (13.3%)	**34 (70.8%)**	<0.001
	Delayed treatment	**23 (76.7%)**	3 (6.3%)	
	Other	3 (10.0%)	11 (22.9%)	
Case-vignette 2	Immediate treatment	0	**23 (47.9%)**	<0.001
	Delayed treatment	**28 (93.3%)**	15 (31.3%)	
	Other	2 (6.7%)	10 (20.8%)	
Case-vignette 3	Immediate treatment	**15 (50.0%)**	**42 (87.5%)**	<0.001
	Delayed treatment	13 (43.3%)	5 (10.4%)	
	Other	2 (6.7%)	1 (2.1%)	
Case-vignette 4	Immediate treatment	**23 (76.7%)**	**46 (95.8%)**	0.016
	Delayed treatment	6 (20.0%)	1 (2.1%)	
	Other	1 (3.3%)	1 (2.1%)	
Case-vignette 5	Immediate treatment	0	**27 (56.3%)**	<0.001
	Delayed treatment	**21 (70.0%)**	11 (22.9%)	
	Other	9 (30.0%)	10 (20.8%)	
Case-vignette 6	Immediate treatment	1 (3.3%)	**17 (35.4%)**	<0.001
	Delayed treatment	**21 (70.0%)**	16 (33.3%)	
	Other	8 (26.7%)	15 (31.3%)	
Case-vignette 7	Immediate treatment	**15 (50.0%)**	**41 (85.4%)**	<0.001
	Delayed treatment	13 (43.3%)	1 (2.1%)	
	Other	2 (6.7%)	6 (12.5%)	
Case-vignette 8	Immediate treatment	**27 (90.0%)**	**48 (100%)**	0.054
	Delayed treatment	3 (10.0%)	0	
	Other	0	0	
Case-vignette 9	Immediate treatment	**26 (86.7%)**	**47 (97.9%)**	0.054
	Delayed treatment	3 (10.0%)	0	
	Other	1 (3.3%)	1 (2.1%)	

Bold numbers indicate the preferred treatment per group.

SUPPLEMENT Table S3. Case vignettes and years of experience as a medical oncologist

Case-vignette	Preferred treatment	5 years or less (n = 8)	6 – 10 years (n = 11)	11 years or more (n = 59)	P value
Case-vignette 1	Immediate treatment	0	**5 (45.5%)**	**33 (55.9%)**	0.004
	Delayed treatment	**7 (87.5%)**	**5 (45.5%)**	14 (23.7%)	
	Other	1 (12.5%)	1 (9.1%)	12 (20.3%)	
Case-vignette 2	Immediate treatment	0	1 (9.1%)	22 (37.3%)	0.042
	Delayed treatment	**7 (87.5%)**	**9 (81.8%)**	**27 (45.8%)**	
	Other	1 (12.5%)	1 (9.1%)	10 (16.9%)	
Case-vignette 3	Immediate treatment	3 (37.5%)	**7 (63.6%)**	**47 (79.7%)**	0.034
	Delayed treatment	**5 (62.5%)**	4 (36.4%)	9 (15.3%)	
	Other	0	0	3 (5.1%)	
Case-vignette 4	Immediate treatment	**6 (75.0%)**	**9 (81.8%)**	**54 (91.5%)**	0.167
	Delayed treatment	2 (25.0%)	1 (9.1%)	4 (6.8%)	
	Other	0	1 (9.1%)	1 (1.7%)	
Case-vignette 5	Immediate treatment	0	1 (9.1%)	**26 (44.1%)**	0.004
	Delayed treatment	**7 (87.5%)**	**7 (63.6%)**	18 (30.5%)	
	Other	1 (12.5%)	3 (27.3%)	15 (25.4%)	
Case-vignette 6	Immediate treatment	0	2 (18.2%)	16 (27.1%)	0.424
	Delayed treatment	**6 (75.0%)**	**6 (54.5%)**	**25 (42.4%)**	
	Other	2 (25.0%)	3 (27.3%)	18 (30.5%)	
Case-vignette 7	Immediate treatment	**4 (50.0%)**	**6 (54.5%)**	**46 (78.0%)**	0.047
	Delayed treatment	**4 (50.0%)**	3 (27.3%)	7 (11.9%)	
	Other	0	2 (18.2%)	6 (10.2%)	
Case-vignette 8	Immediate treatment	**7 (87.5%)**	**10 (90.9%)**	**58 (98.3%)**	0.145
	Delayed treatment	1 (12.5%)	1 (9.1%)	1 (1.7%)	
	Other	0	0	0	
Case-vignette 9	Immediate treatment	**7 (87.5%)**	**10 (90.9%)**	**56 (94.9%)**	0.302
	Delayed treatment	1 (12.5%)	1 (9.1%)	1 (1.7%)	
	Other	0	0	2	

Bold numbers indicate the preferred treatment per group.

SUPPLEMENT File S1. Survey

General questions

- In which country do you work?
    - Open question

- Wat is your age?
    - Open question

- What is your gender?
    - Female
    - Male

- In what type of hospital do you work?
    - Academic hospital that performs pancreatic surgery
    - Academic hospital that does not perform pancreatic surgery
    - Teaching hospital (non-academic) that performs pancreatic surgery
    - Teaching hospital (non-academic) that does not perform pancreatic surgery
    - Other, namely

- How many years have you been registered as an oncologist?
    - 5 years or less
    - 6-10 years
    - 11 years or more

- How many patients with pancreatic cancer do you personally **treat** with systemic therapy (all stages):
    - Never
    - 10 patients or less per year
    - 11-20 patients per year
    - 21-50 patients per year
    - 51-100 patients per year
    - 101 patients per year or more

- In patients with metastatic pancreatic cancer, in general, which systemic therapy do you prefer:
  - FOLFINIRNOX (or modified versions)
  - Gemcitabine plus nab-paclitaxel
  - Gemcitabine monotherapy
  - Clinical trials

- How many patients do you personally see in your outpatient clinic with asymptomatic metastastatic pancreatic cancer?
  - Never
  - 10 patients or less per year
  - 11-20 patients per year
  - 21 patients per year or more

- In the hospital you work at, is there a form of structured follow-up (e.g., periodic imaging) after curative treatment
  - No
  - Only within trials
  - Yes

<u>Attitude towards treating asymptomatic patients with systemic therapy</u>

- In your opinion, what is the best time point to start with systemic treatment in a patient with asymptomatic metastatic pancreatic cancer that visits your outpatient clinic directly after the diagnosis of metastatic disease:
  - Immediately after diagnosis
  - Once symptoms occur
  - In case of objective (radiological, tumor marker) signs of disease progression after the first diagnosis of metastatic disease
  - Once symptoms occur or in case of objective signs of disease progression, whichever comes first.
  - Other, namely

- Do financial incentives play a role in your decision when to start with systemic treatment in patients with asymptomatic metastatic pancreatic cancer (i.e., treating patients immediately after diagnosis or at symptoms/objective signs of disease progression)?

  - Yes
  - No

- If financial incentives play a role in your decision, please explain the reason why
  - Open question

- In your opinion, is a randomized trial justified to assess optimal timing of start with systemic therapy in patients with asymptomatic metastatic pancreatic cancer?
  - Yes
  - No

<u>Case vignettes:</u>

You are presented with eight case vignettes. All cases are highly similar and each case only differs in one aspect from the others. This difference has been made visible in “bold” print. We are interested to learn about your personal advice for this patient in your consultation room.

Case 1:
A 60-year-old patient without significant comorbidities, WHO 0, one year after pancreatoduodenectomy (PD) for pancreatic ductal adenocarcinoma (PDAC). During routine follow-up after surgery **1 liver metastasis** is seen on the CT-scan. Pathology confirms metastatic pancreatic cancer. Patient has no complaints. What would you advise?

A) Direct treatment: start directly with chemotherapy
B) Delayed treatment: wait with chemotherapy until complaints
C) Delayed treatment: wait with chemotherapy until disease progression on imaging
D) Delayed treatment: wait with chemotherapy until complaints or disease progression on imaging, whichever comes first.
E) Other, namely

Case 2:
A 60-year-old patient without significant comorbidities, WHO 0, one year after pancreatoduodenectomy (PD) for pancreatic ductal adenocarcinoma (PDAC). During routine follow-up after surgery **1 small lung metastasis** is seen on the CT-scan. Pathology confirms that it is metastatic pancreatic cancer. Patient has no complaints. What would you advise?

A) Direct treatment: start directly with chemotherapy
B) Delayed treatment: wait with chemotherapy until complaints
C) Delayed treatment: wait with chemotherapy until disease progression on imaging
D) Delayed treatment: wait with chemotherapy until complaints or disease progression on imaging, whichever comes first.
E) Other, namely

Case 3:

A 60-year-old patient without significant comorbidities, WHO 0, one year after pancreatoduodenectomy (PD) for pancreatic ductal adenocarcinoma (PDAC). During routine follow-up after surgery **6 lung metastases** are seen on the CT-scan. Pathology confirms that it is metastatic pancreatic cancer. Patient has no complaints. What would you advise?

A) Direct treatment: start directly with chemotherapy
B) Delayed treatment: wait with chemotherapy until complaints
C) Delayed treatment: wait with chemotherapy until disease progression on imaging
D) Delayed treatment: wait with chemotherapy until complaints or disease progression on imaging, whichever comes first.
E) Other, namely

Case 4:

A 60-year-old patient without significant comorbidities, WHO 0, one year after pancreatoduodenectomy (PD) for pancreatic ductal adenocarcinoma (PDAC). During routine follow-up after surgery **3 liver metastases and 3 lung metastases** are seen on the CT-scan. Pathology confirms that it is metastatic pancreatic cancer. Patient has no complaints. What would you advise?

A) Direct treatment: start directly with chemotherapy
B) Delayed treatment: wait with chemotherapy until complaints
C) Delayed treatment: wait with chemotherapy until disease progression on imaging
D) Delayed treatment: wait with chemotherapy until complaints or disease progression on imaging, whichever comes first.
E) Other, namely

Case 5:

An **80-year-old patient** without significant comorbidities, WHO 0, one year after pancreatoduodenectomy (PD) for pancreatic ductal adenocarcinoma (PDAC). During routine follow-

up after surgery **1 liver metastasis** is seen on the CT-scan. Pathology confirms metastatic pancreatic cancer. Patient has no complaints. What would you advise?

A) Direct treatment: start directly with chemotherapy
B) Delayed treatment: wait with chemotherapy until complaints
C) Delayed treatment: wait with chemotherapy until disease progression on imaging
D) Delayed treatment: wait with chemotherapy until complaints or disease progression on imaging, whichever comes first.
E) Other, namely

Case 6:

A 60-year-old patient one year after pancreatoduodenectomy (PD) for pancreatic ductal adenocarcinoma (PDAC). **Patient has significant co-morbidities: type 2 diabetes and heart failure NYHA class 2, due to the comorbidities the WHO performance status is 2**. During routine follow-up after surgery 1 liver metastasis is seen on the CT-scan. Pathology confirms metastatic pancreatic cancer. Patient has no complaints. What would you advise?

A) Direct treatment: start directly with chemotherapy
B) Delayed treatment: wait with chemotherapy until complaints
C) Delayed treatment: wait with chemotherapy until disease progression on imaging
D) Delayed treatment: wait with chemotherapy until complaints or disease progression on imaging, whichever comes first
E) Other, namely

Case 7:

A 60-year-old patient without significant comorbidities, WHO 0. **No history of pancreatic cancer. By chance, during an abdominal ultrasound, an abnormality is seen in the liver for which further analysis takes place.** Pathology confirms metastatic pancreatic cancer. Patient has no complaints. What would you advise?

A) Direct treatment: start directly with chemotherapy
B) Delayed treatment: wait with chemotherapy until complaints
C) Delayed treatment: wait with chemotherapy until disease progression on imaging
D) Delayed treatment: wait with chemotherapy until complaints or disease progression on imaging, whichever comes first
E) Other, namely

Case 8:

A 60-year-old patient without significant comorbidities, WHO 0, one year after pancreatoduodenectomy (PD) for pancreatic ductal adenocarcinoma (PDAC). During routine follow-up after surgery 2 liver metastases are seen on the CT-scan. Pathology confirms metastatic pancreatic cancer.

You have decided to wait with chemotherapy until complaints. After 6 weeks a new CT scan has been made. CT scan shows progression of disease, patient now has **6 liver metastases.** Patient still has no complaints and feels well. What would you advise?

A) Start directly with chemotherapy
B) Delayed treatment: start with chemotherapy once symptoms occur
C) Delayed treatment: start chemotherapy once objective (radiological, tumor marker) signs of disease progression after the first diagnosis of metastatic disease occur
D) Delayed treatment: start with chemotherapy once symptoms occur or in case of objective signs of disease progression, whichever comes first.
E) Other, namely….

Case 9:

A 60-year-old patient without significant comorbidities, WHO 0, one year after pancreatoduodenectomy (PD) for pancreatic ductal adenocarcinoma (PDAC). During routine follow-up after surgery 2 liver metastases are seen on the CT-scan. Pathology confirms that it is metastatic pancreatic cancer.

You have decided to wait with chemotherapy until complaints. After 8 weeks a new CT scan has been made. CT scan shows progression of disease, patient now has **3 liver and 3 lung metastases.** Patient still has no complaints and feels well. What would you advise?

A) Start directly with chemotherapy
B) Delayed treatment: start with chemotherapy once symptoms occur
C) Delayed treatment: start chemotherapy once objective (radiological, tumor marker) signs of disease progression after the first diagnosis of metastatic disease occur
D) Delayed treatment: start with chemotherapy once symptoms occur or in case of objective signs of disease progression, whichever comes first.
E) Other, namely….

End of survey:

Thank you for participating in our study. If you have any questions regarding this survey, you can get in touch with Simone Augustinus (s.augustinus@amsterdamumc.nl), PhD-candidate at Amsterdam UMC. Or leave a remark below

- Open question

The results obtained by this survey will be used for future (prospective) studies on patients diagnosed with pancreatic cancer. As stated previously, your emailaddress will be stored separately from the answers given. Do you want to be contacted by the research team for (participating in) future studies on pancreatic cancer?

- Yes
- No
